# What does a good RCT recruitment consultation look like? A new simple six-step model to promote information sharing and recruitment to RCTs

**DOI:** 10.1186/1745-6215-16-S2-O21

**Published:** 2015-11-16

**Authors:** Alba Realpe, Ann Adams, Peter Wall, Damian Griffin, Jenny L Donovan

**Affiliations:** 1 University of Warwick, Coventry, UK; 2 University of Bristol, Bristol, UK

## Objective

The mode of delivery of trial information is a key determinant of recruitment to randomised controlled trials (RCTs), which can be modified in order to encourage patients to participate. This paper presents the development and initial validation of a simple six-step model to support recruitment.

## Study design and setting

92 recruitment consultations with 60 new patients were recorded and analysed during a pilot RCT comparing surgical and non-surgical interventions for hip impingement. Recordings were analysed using techniques of thematic analysis and focused conversation analysis pioneered in previous studies. Analysis of recordings continued during the full-scale trial.

## Results

The pilot study was successful, with 70% of patients approached across 9 centres agreeing to take part in the RCT, and the full-scale trial has achieved 75% recruitment in 19 centres. A simple six-step model providing a framework for good recruitment practice was developed at the pilot phase and then validated, and tested in the main trial. . The model enabled recruiters to explain the design and conduct of the RCT and provide reassuring information for patients in the context of consultations very different from routine practice.

## Conclusion

The six-step model provides a useful framework for recruitment to RCTs. It encourages the implementation of good RCT recruitment practice and provides strategies to support recruiters. The model requires further testing in a wide range of RCTs and clinical contexts.

